# Dedifferentiation of Human Primary Thyrocytes into Multilineage Progenitor Cells without Gene Introduction

**DOI:** 10.1371/journal.pone.0019354

**Published:** 2011-04-27

**Authors:** Keiji Suzuki, Norisato Mitsutake, Vladimir Saenko, Masatoshi Suzuki, Michiko Matsuse, Akira Ohtsuru, Atsushi Kumagai, Tatsuya Uga, Hiroshi Yano, Yuji Nagayama, Shunichi Yamashita

**Affiliations:** 1 Department of Molecular Medicine, Atomic Bomb Disease Institute, Nagasaki University Graduate School of Biomedical Sciences, Nagasaki, Nagasaki, Japan; 2 Department of International Health and Radiation Research, Atomic Bomb Disease Institute, Nagasaki University Graduate School of Biomedical Sciences, Nagasaki, Nagasaki, Japan; 3 Department of Medical Gene Technology, Atomic Bomb Disease Institute, Nagasaki University Graduate School of Biomedical Sciences, Nagasaki, Nagasaki, Japan; 4 Takashi Nagai Memorial International Hibakusha Medical Center, Nagasaki University Hospital, Nagasaki, Nagasaki, Japan; 5 Department of Transplantation and Digestive Surgery, Nagasaki University Hospital, Nagasaki, Nagasaki, Japan; 6 Division of Surgical Oncology, Department of Translational Medical Sciences, Nagasaki University Hospital, Nagasaki, Nagasaki, Japan; 7 Nagasaki University Research Centre for Genomic Instability and Carcinogenesis (NRGIC), Nagasaki, Nagasaki, Japan; Wellcome Trust Centre for Stem Cell Research, United Kingdom

## Abstract

While identification and isolation of adult stem cells have potentially important implications, recent reports regarding dedifferentiation/reprogramming from differentiated cells have provided another clue to gain insight into source of tissue stem/progenitor cells. In this study, we developed a novel culture system to obtain dedifferentiated progenitor cells from normal human thyroid tissues. After enzymatic digestion, primary thyrocytes, expressing thyroglobulin, vimentin and cytokeratin-18, were cultured in a serum-free medium called SAGM. Although the vast majority of cells died, a small proportion (∼0.5%) survived and proliferated. During initial cell expansion, thyroglobulin/cytokeratin-18 expression was gradually declined in the proliferating cells. Moreover, sorted cells expressing thyroid peroxidase gave rise to proliferating clones in SAGM. These data suggest that those cells are derived from thyroid follicular cells or at least thyroid-committed cells. The SAGM-grown cells did not express any thyroid-specific genes. However, after four-week incubation with FBS and TSH, cytokeratin-18, thyroglobulin, TSH receptor, PAX8 and TTF1 expressions re-emerged. Moreover, surprisingly, the cells were capable of differentiating into neuronal or adipogenic lineage depending on differentiating conditions. In summary, we have developed a novel system to generate multilineage progenitor cells from normal human thyroid tissues. This seems to be achieved by dedifferentiation of thyroid follicular cells. The presently described culture system may be useful for regenerative medicine, but the primary importance will be as a tool to elucidate the mechanisms of thyroid diseases.

## Introduction

Adult stem cells are rare, premature cells capable of self-renewal and generating distinct differentiated cell types within a tissue. There is no doubt that identification and isolation of stem cells have potentially important implications not only for regenerative medicine but for understanding of pathogenesis of a variety of diseases.

The existence of stem cells in adult human thyroid tissue has been suggested by several studies. Based on immunohistochemical analyses, two groups proposed that p63-positive cells residing in solid cell nests may be undifferentiated stem cells and undergo follicular maturation [1], [2], [3], [4]. Thomas et al. found that a small subset of cells in nodular goiters express a stem cell marker *Oct-4* and endodermal markers *GATA-4* and *HNF4α* using RT-PCR, immunohistochemistry and flow cytometry; however, no functional characterization was performed [5].

The subsequent work by the same group (Lan et al.) reported that possible stem cells were isolated from nodular goiters using a sphere formation approach in a serum-free medium containing epidermal growth factor (EGF) and basic fibroblast growth factor (bFGF), and some functional studies were performed [6]. This method has been frequently used to isolate stem cells from several other tissues. The obtained sphere cells did not express thyroid specific genes such as *thyroglobulin (TG)* and *TSH receptor (TSH-R)*, but their expressions emerged in monolayer culture stimulated by serum and TSH. However, the growth potential of the cells was very limited: the proliferation stopped after 4–5 days of culture.

More recently, Fierabracci et al. have also reported the identification of stem/progenitor cells in normal human thyroid tissues [7]. They also used similar approach with serum-free/EGF/bFGF medium and sphere formation. The isolated cells were probably not fully undifferentiated or not homogeneous, since TG or thyroid peroxidase (TPO) expression was in part detected. After stimulation with serum, the cells formed follicle-like structure in collagen gel and produced T_4_. Surprisingly, some isolated cells underwent multilineage differentiation into neurogenic and adipogenic lineages.

So far, two above-mentioned studies described isolation and functional characterization of possible stem cells from human thyroid tissues (including goiters). However, there are still some uncertainties as for origin and homogeneity of the cells. In addition, Lan et al. used cells released by enzymatic digestion, whereas Fierabracci et al. used residual thyroid fragments, presumably containing cells tightly bound to collagen fibers, regardless of using very similar medium. Therefore, the isolated cells by Lan et al. may be different from those by Fierabracci et al. We tried both methods but could not reproduce the sphere formation in our hands (unpublished data). Thus, the origin and procedure of isolation of thyroid stem cells still remain obscure.

Recent reports have provided another clue to gain insight into source of primitive stem/progenitor cells. In 2007, induced pluripotent stem (iPS) cells were established from adult human fibroblasts by direct reprogramming with defined factors [8]. Recently, not only reprogramming (generating pluripotent stem cells) but also generating tissue stem/progenitor cells (i.e. dedifferentiation or partial reprogramming) has also been reported. Mani et al. have demonstrated that differentiated mammary epithelial cells can be converted into mammary tissue stem cells by introducing genes related to epithelial-mesenchymal transition (EMT) [9]. Moreover, cancer stem cells (tumor-initiating cells) have also been generated from cancer cells [10] or even from normal epithelial cells [11]. These reports suggest that tissue stem/progenitor cells could be generated from mature and differentiated cells.

In the present study, we present a novel culture system in which progenitor cells can be emerged and propagated from normal human thyroid follicular cells by dedifferentiation. We also demonstrate that the cells possess multilineage differentiation potential.

## Materials and Methods

### Cell culture and reagents

We used anaplastic thyroid carcinoma cell line: FRO; papillary thyroid carcinoma cell lines: TPC-1 and KTC-1; follicular thyroid carcinoma cell line: WRO. All these cells were of human origin. FRO and WRO were kindly provided by Dr G. Juillard (University of California, Los Angeles, CA). TPC-1 and KTC-1 were kindly provided by Dr Sato (Kanazawa University, Kanazawa, Japan) and Dr Kurebayashi (Kawasaki Medical School, Kurashiki, Japan), respectively. All the cells were maintained in RPMI-1640 medium supplemented with 5% fetal bovine serum (FBS; Invitrogen, Carlsbad, CA) and penicillin/streptomycin (Sigma, St. Louis, MO) at 37°C in a humidified atmosphere with 5% CO_2_. Primary human thyroid cells (PT) were isolated from thyroid tissues obtained during subtotal thyroidectomy in patients with Graves' disease as described previously [12] and cultured overnight in DMEM:F12 medium (1∶2) supplemented with 3.3% FBS and penicillin/streptomycin (PT medium). All experiments were performed after obtaining approval by the Ethical Committee of Nagasaki University Graduate School of Biomedical Sciences. Written informed consent was obtained from each individual. SAGM was purchased from Lonza (Basel, Switzerland). For neurogenic and adipogenic differentiation, Human Neural Stem Cell Functional Identification Kit (R&D Systems, Minneapolis, MN) and StemPro Adipogenesis Differentiation Kit (Invitrogen) were used, respectively. Bovine TSH (bTSH) was purchased from Sigma. Oil-red-O staining was performed using Lipid Assay kit (Primary Cell Co., Sapporo, Japan).

### Immunofluorescence

Cells cultured on coverslips were fixed and immunolabeled as previously described [13]. The following primary antibodies were used: anti-phosphorylated H2AX (Ser139) (mouse clone JBW301, Millipore, Billerica, MA), anti-cytokeratin-18 (Clone DC 10, Dako Cytomation, Glostrup, Denmark), anti-vimentin (Clone V9, Dako Cytomation), anti-STRO-1 (Clone STRO-1, R&D Systems) and anti-TG (mouse clone B34.1, GeneTex, Irvine, CA). Anti-β-III-tubulin antibody was included in Human Neural Stem Cell Functional Identification Kit (R&D Systems). After labeling with secondary antibodies conjugated with Alexa 488 or 594 (Invitrogen), images were acquired using a fluorescence microscopy DM6000B (Leica Microsystems, Tokyo, Japan).

### Senescence-associated β-galactosidase (SA-β-gal) staining

SA-β-gal staining was carried out as described by Dimri et al. [14]. Briefly, cells were fixed with 2% paraformaldehyde solution containing 0.2% glutaraldehyde for 5 min at room temperature. After fixation, the cells were then incubated with stain solution (40 mM citric acid/sodium phosphate, pH 6.0, 5 mM potassium ferrocyanide, 5 mM potassium ferricyanide, 150 mM NaCl, and 2 mM MgCl_2_) containing 1 mg/ml X-gal at 37°C for overnight.

### Fluorescence-activated cell sorting

Isolated PT were cultured overnight in PT medium supplemented with 10 mIU/ml bTSH. On the next day, PT were collected and incubated with anti-STRO-1 or anti-TPO (Clone MoAb47, Dako Cytomation) antibody. After labeling with secondary antibody conjugated with Alexa 488 and 7-AAD (BD Biosciences, Franklin Lakes, NJ), the cells were analyzed and sorted using a FACS Vantage SE (BD Biosciences).

### Real-time RT-PCR

Total RNA was extracted using ISOGEN reagent (Nippon gene, Tokyo, Japan) and reverse transcribed using SuperScript III First-Strand Synthesis SuperMix (Invitrogen) with random hexamers. The following PCR amplifications were performed using QuantiTect SYBR Green RT-PCR kit (Qiagen, Valencia, CA) for TTF1 and SYBR Premix Ex Taq Perfect Real Time kit (TaKaRa Bio, Ohtsu, Japan) for other genes in a Thermal Cycler Dice Real Time System (TaKaRa Bio). The cycle threshold value, which was determined using second derivative, was used to calculate the normalized expression of the indicated genes using Q-Gene software [15], using ribosomal RNA 18S as a reference. The following primer pairs were used: 18S, 5′-GTAACCCGTTGAACCCCATT-3′ and 5′-CCATCCAATCGGTAGTAGCG-3′; TG, 5′-CTGGTGTGTCATGGACAGCGGAGAA-3′ and 5′-CCCGAGATTGTCTCACACAGGAT-3′; TSH-R 5′-CTATAGATGTGACTCTGCAGCAGCT-3′ and 5′-GAGGGCATCAGGGTCTATGTAAGT-3′; PAX8 5′-CCCCCTACTCCTCCTACAGC-3′ and 5′-ACTGTCCCCATGGCAACTAC-3′; TTF1 5′-CCATGAGGAACAGCGCCTC-3′ and 5′-CTCACG TCCCCCAGCGA-3′.

### Microarray analysis

Total RNAs were extracted from PT from three different samples (PT0808, PT0811 and PT0812) on the next day of initial plating and corresponding SAGM-grown lines at two weeks after plating using ISOGEN reagent (Nippon gene) and subjected to Affymetrix GeneChip Human Genome U133 Plus 2.0 microarray analysis service (Bio Matrix Research, Nagareyama, Japan). The array contained 54,675 probes for mRNAs. GeneSpring Software (Agilent Technologies, Santa Clara, CA) was used for data analyses. The data were deposited in NCBI GEO site (accession number: GSE24553).

## Results

### Isolation of proliferative cells from normal human thyroid tissues

In an attempt to isolate primitive human thyroid epithelial cells, we tried several commercially available media containing growth factors but not serum which is generally thought to enforce differentiation program. Those were basically developed for growing human primary cells or tissue stem cells. Among them, we found that SAGM was the most suitable for our purpose, which was developed for growing human primary small airway epithelial cells.

**Figure 1:**
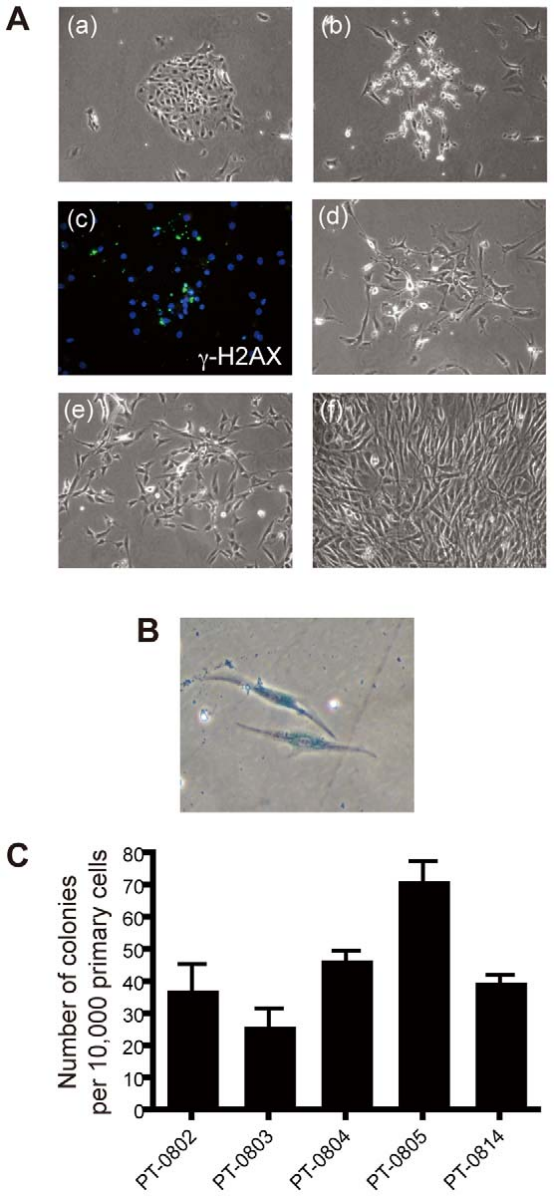
Isolation of proliferative cells from normal human thyroid tissue. A, (a) PT on the next day of plating. (b) A cluster of dead cells within 7–10 days. (c) γ-H2AX staining of dead cells. (d–f) Expansion of cells after 7–10 days. B, After cultured for three months, the cells showed positive for SA-β-gal staining. C, Clonogenic ability of the primary cells incubated with SAGM. Each bar indicates the mean and SD of three dishes.

After enzymatic digestion of thyroid tissues, primary thyrocytes (PT) were plated in PT medium containing FBS which facilitated attachment of the cells. On the next day, the PT medium was replaced by SAGM (Figure 1A; a). Within 7–10 days culture, the vast majority of the cells died (Figure 1A; b). This was probably due to induction of apoptosis, which was confirmed by nuclear fragmentation and γ-H2AX staining (massive DNA double strand breaks, Figure 1A; c) [16]. By contrast, clonal expansion of a small proportion became evident by 7–10 days culture, and they grew continuously (Figure 1A; d–f). These cells showed mesenchymal cell-like morphology (Figure 1A; e, f). Doubling time of the cells was approximately 24–36 hours during first 1–2 months. Time-lapse analysis looking at cell cycle length demonstrated that some of them showed asymmetric division in culture (Figure S1). The SAGM-grown cells had 46 chromosomes with no apparent gross rearrangement by Giemsa staining. Growth rate of the cells gradually declined and eventually became flat by approximately 3–4 months. At this point, the cells were positive for SA-β-gal staining, indicating that they became senescent (Figure 1B).

We used more than 10 tissues from Graves' patients and successfully obtained proliferative cells from all of them. On average, approximately 40–50 cells were estimated to have clonogenic potential among 10,000 PT (Figure 1C).

### Characteristic expression of intermediate filaments in thyroid cells

**Figure 2:**
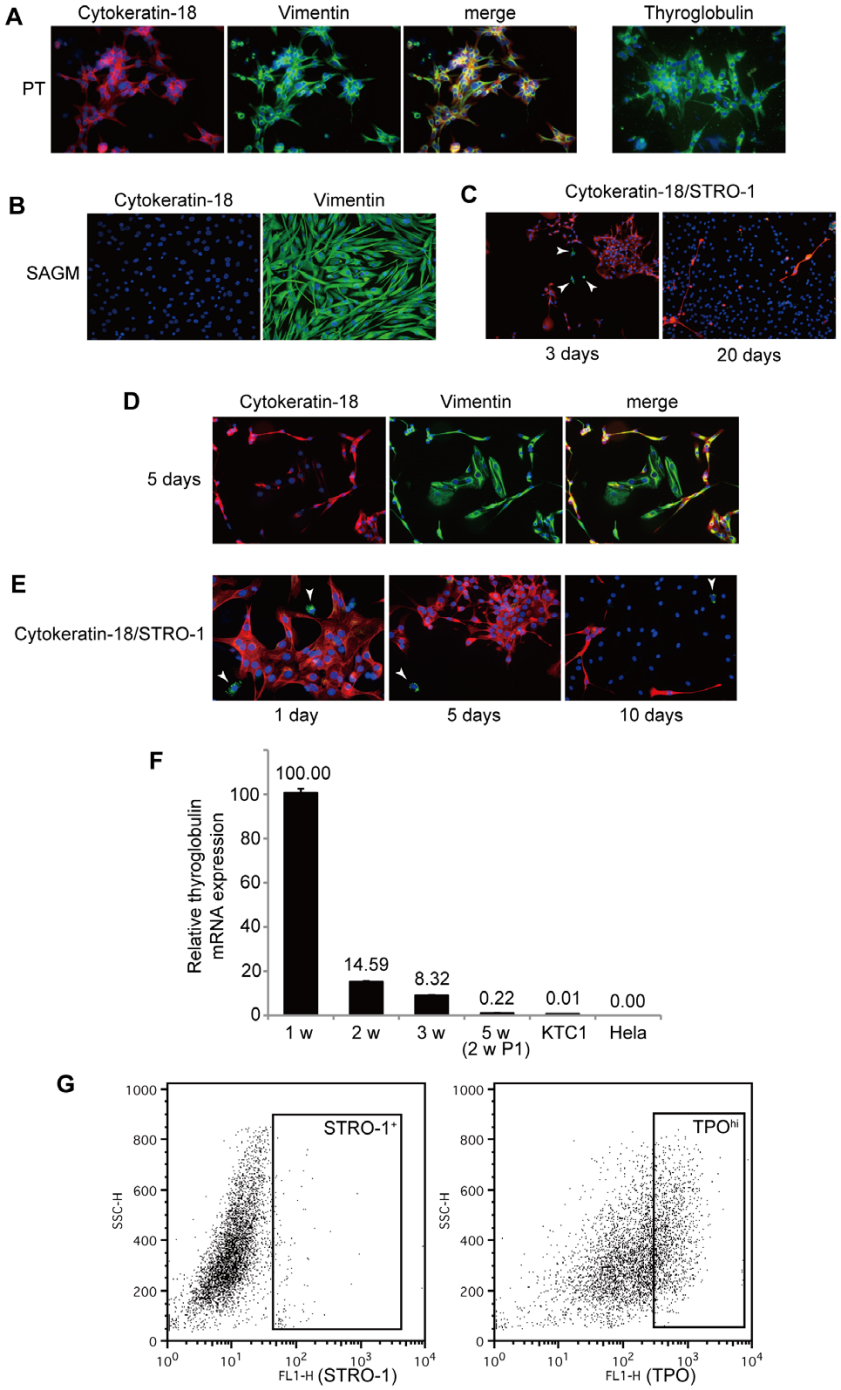
Origin of the SAGM-grown cells. A, On the next day after plating, PT were fixed and followed by immunofluorescence staining using following primary antibodies: cytokeratin-18 (red), vimentin (green) and TG (green). B, After grown for three weeks, the SAGM-grown cells were fixed and stained for cytokeratin-18 (red) and vimentin (green). C and E, Cells were fixed and then stained for STRO-1 (green) and cytokeratin-18 (red) on indicated days after plating. Arrowheads indicate STRO-1-positive cells. D, Five days after plating, cells were fixed and stained for cytokeratin-18 (red) and vimentin (green). At the center of each image, cell cluster just starting expansion is shown. F, At indicated time points, total RNA was extracted and then subjected to qRT-PCR for *TG*. Each bar indicates the mean and SD of the data collected in triplicate. 2 w P1: two weeks after first passage (the cells were subcultured at three weeks). RNAs from KTC-1 and Hela cells were also used as controls. Similar results were obtained in three different samples. G, On the next day after plating, PT were stained with anti-STRO-1 or anti-TPO antibody and subjected to FACS. Each box means sorting gate for STRO-1-positive or TPO^hi^ cells.

To characterize the SAGM-grown cells, we investigated expression of intermediate filaments because their expression pattern depends on type of cell lineage. First of all, we checked the expression in PT. As shown in Figure 2A, cytokeratin-18, generally found in single layer epithelial tissues, was abundantly expressed in PT. Expression of vimentin, which is usually found in non-epithelial type of cells such as fibroblasts and endothelial cells, was also observed in PT (Figure 2A). TG expression was confirmed, ensuring that these cells are *bona fide* thyroid follicular cells (Figure 2A). Next, we checked the cytokeratin-18 and vimentin expression in thyroid cancer cell lines. FRO, TPC-1 and WRO expressed both filaments as well as PT (data not shown). These data suggest that both cytokeratin-18 and vimentin are expressed in normal thyroid and thyroid cancer cells. We then examined the SAGM-grown cells and found that only vimentin but not cytokeratin-18 was expressed in these cells (Figure 2B).

### Origin of the SAGM-grown cells

The above observations prompted us to explore the origin of the SAGM-grown cells because all examined thyroid cells even anaplastic cancer cell line expressed cytokeratin-18. Since vimentin is used as a marker of mesenchymal cells, we next used a mesenchymal stem cell marker STRO-1. Three days after plating (no dead cells at this time point), a small number of cells were positive for STRO-1, and these cells were negative for cytokeratin-18 (Figure 2C, arrowheads). We found that all cytokeratin-18-negative cells were STRO-1/vimentin-positive. This was confirmed by analyzing about 4,500 cells by fluorescence microscopy (Table 1). Vimentin was expressed in all attached cells (Table 1). These data indicate that there were two types of attached cells in the culture at this time point: cytokeratin-18/vimentin double-positive cells and vimentin/STRO-1 double-positive cells. By contrast, the SAGM-grown cells (20 days after plating) did not express STRO-1 (Figure 2C).

**Table 1:** Expression of lineage-specific markers in primary thyrocytes.

Cells	Total count	Cyt^**+**^	Vim^**+**^	Cyt^**+**^/Vim^**+**^	Vim^**+**^/STRO-1^**+**^
PT-0808	4507	4472 (99.2)	4507 (100)	4472 (99.2)	35 (0.8)
PT-0811	4856	4809 (99.0)	4856 (100)	4472 (99.0)	47 (1.0)
PT-0818	4728	4686 (99.1)	4728 (100)	4686 (99.1)	42 (0.9)
PT-0901	4413	4376 (99.2)	4413 (100)	4376 (99.2)	37 (0.8)

No. of cells (%).

Determined by immunofluorescence.

Next, we carefully investigated the process of the beginning of proliferation by fixing cells every two days. We found clusters with 5–10 cells just starting expansion 5–7 days after plating (Figure 2D). As expected, vimentin was expressed in these cells, while cytokeratin-18 was only weakly expressed in the same cells (Figure 2D), suggesting that the proliferating cells were losing cytokeratin-18 expression. In contrast, at the same time point, vimentin/STRO-1-positive and cytokeratin-18-negative cells did not proliferate at all (Figure 2E, arrowheads; Table S1).

We also measured relative expression of *TG* mRNA by real-time quantitative RT-PCR (qRT-PCR). At one week, *TG* expression was due to residual differentiated thyroid follicular cells (Figure 2F). The amount of *TG* mRNA gradually decreased; however, the SAGM-grown cells after three-week culture still expressed low level of *TG* mRNA, which was still higher than that in KTC-1 cells (Figure 2F). KTC-1 cells show relatively higher *PAX-8* and *TTF-1* transcripts among thyroid cancer cell lines [17].

To further confirm the origin of the proliferating cells, we next performed selective cell culture in SAGM after fluorescence-activated cell sorting (FACS) using anti-STRO-1 (for sorting STRO-1-positive cells) and anti-TPO (for sorting thyroid follicular cells) antibodies (Figure 2G). The percentages of TPO-positive cells were 70–90% depending on samples, and we sorted top 40% (TPO^hi^ cells) for subsequent cultures. As expected, STRO-1-positive (approximately 1%) cells did not grow at all in SAGM, whereas TPO^hi^ cells gave rise to proliferating colonies (Table S2).

Taken together, these results suggest that the SAGM-grown cells were derived from thyroid follicular cells or at least thyroid-committed cells.

### Differentiation of the SAGM-grown cells

**Figure 3:**
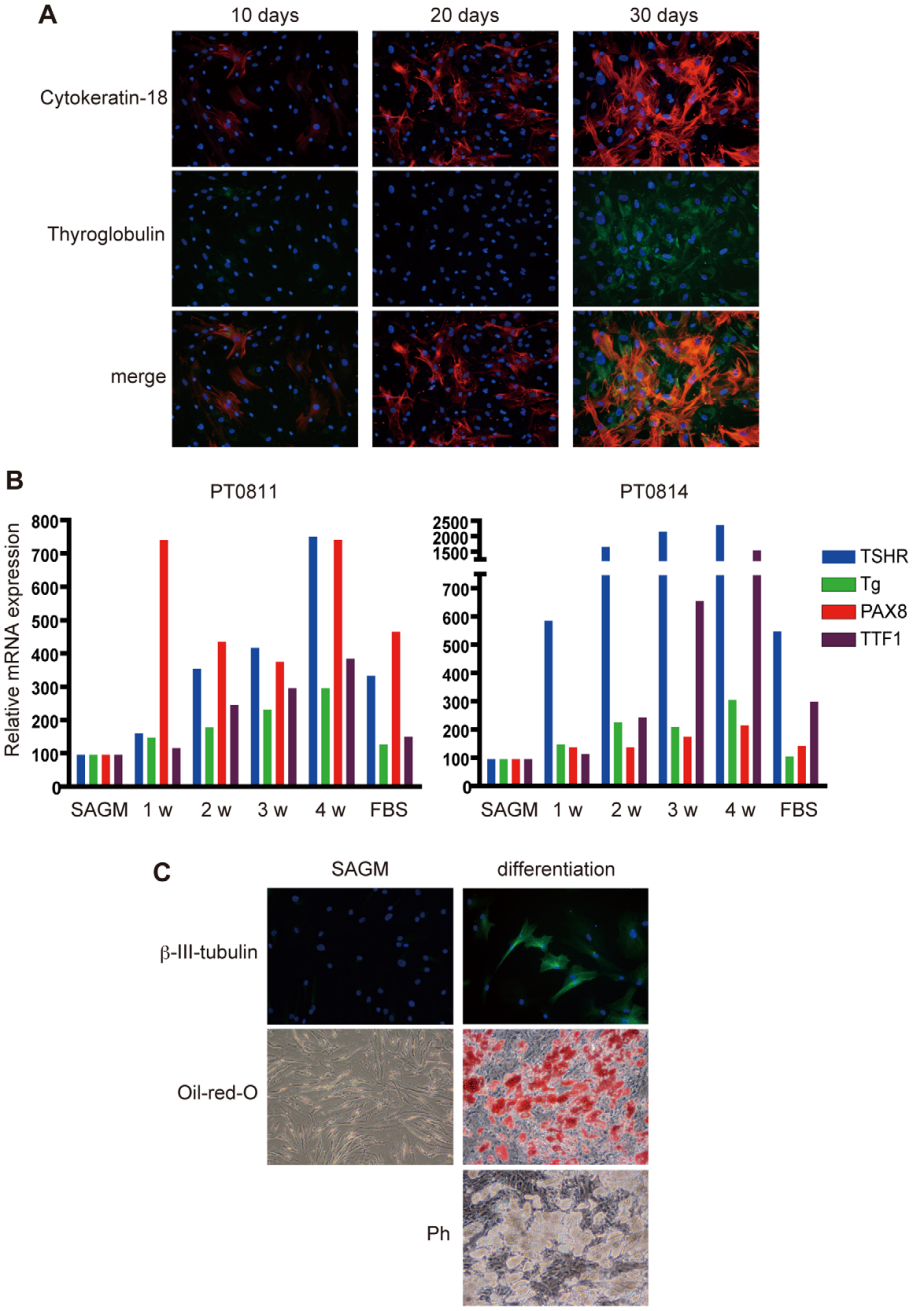
Differentiation of the SAGM-grown cells. A, The SAGM-grown cells were incubated with PT medium supplemented with bTSH for indicated times. The cells were then fixed and stained for cytokeratin-18 (red) and TG (green). B, At indicated time points, total RNA was extracted and subjected to qRT-PCR for indicated gene expressions. Each bar indicates the mean of the data collected in duplicate. C, The SAGM-grown cells were incubated with neurogenic differentiation medium or adipogenic differentiation medium for four weeks. The cells were fixed and stained for β-III-tubulin (upper, immunofluorescence) or lipid (middle, oil-red-O staining). Ph: phase contrast image of cells with lipid droplets. The data are representative of three independent experiments.

The SAGM-grown cells were incubated with PT medium supplemented with 10 mIU/ml bTSH, and the expression of cytokeratin-18 and TG was examined at different time points. The growth of the cells stopped in this medium. As shown in Figure 3A, the expression of cytokeratin-18 was gradually increased (Figure 3A). TG expression was also evident after 30 days stimulation (Figure 3A). We also checked mRNA expression of *TSH-R*, *TG*, *PAX8* and *TTF-1* by qRT-PCR. Although the levels of induction varied depending on the samples, all of the messages were gradually increased (Figure 3B). Interestingly, serum stimulation alone (without bTSH) up-regulated *TSH-R* expression, whereas *TG* induction needed both serum and bTSH stimulation (Figure 3B). These data indicate that the SAGM-grown cells were differentiated into thyroid follicular lineage. We next explored the differentiation potential of the cells into other lineages. Surprisingly, after incubation with the neurogenic differentiating medium for four weeks, the SAGM-grown cells expressed β-III-tubulin, which is a microtubule element of the tubulin family found almost exclusively in neurons (Figure 3C). Moreover, after four-week-treatment with the adipogenic differentiating medium, many lipid droplets were formed, and they were all positive for oil-red-O staining (Figure 3C). We measured the proportion of differentiation marker-positive cells in each differentiating condition. In thyroid differentiation, most of cells (>90%) were positive for TG, while β-III-tubulin-positive and oil-red-O-positive cells varied (48–87%) presumably depending on conditions (Table 2). These data suggest that the SAGM-grown cells have multipotent (at least dipotent) differentiation potential.

**Table 2:** Percentage of differentiated marker-positive cells in each differentiating condition.

Cells	Tg^**+**^	β-III-tub^**+**^	Oil-red-O^**+**^
PT-0808	96.8	67.1	87.0
PT-0902	93.9	55.0	48.6

% of positive cells.

### Gene expression profile of the SAGM-grown cells

**Figure 4:**
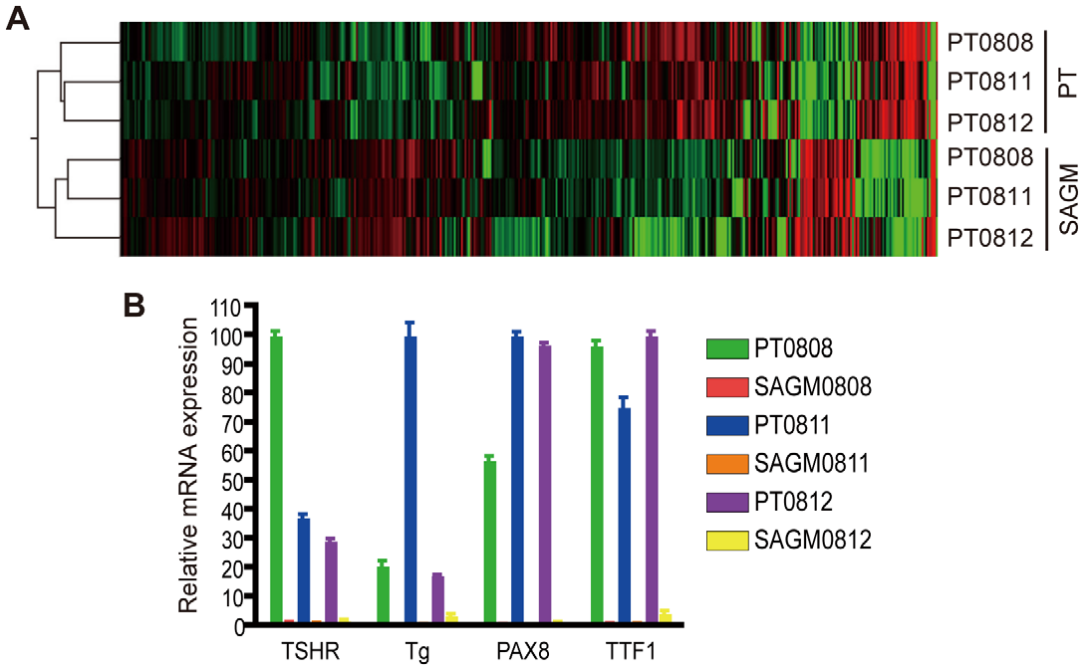
Microarray analysis of PT and corresponding SAGM-grown cells. A, The tree view of hierarchical clustering. PT: total RNA was extracted from PT on the next day of plating. SAGM: the SAGM-grown cells were expanded for two weeks and subcultured. On the next day, total RNA was extracted. The total RNAs were subjected to Affymetrix GeneChip Human Genome U133 Plus 2.0 microarray analysis service. Hierarchical clustering (by Manhattan distance) was performed using probes with “present call” only. Red columns represent higher expression, and green indicate lower expression. B, Confirmation of microarray data. Total RNA was subjected to qRT-PCR for indicated genes. Each bar indicates the mean and SD of the data collected in triplicate.

To perform a comprehensive analysis of differential gene expressions between PT and SAGM-grown cells, we used oligonucleotide-based DNA microarrays, GeneChip Human Genome U133 Plus 2.0 array (Affmetrix). This array system utilizes flag score (present, marginal and absent) calculated by the difference in signals between perfect match (PM) and mismatch (MM) probes. Probes with absent call likely represent undetectably low expression (but not always), and therefore, the fold-change is not accurate. Of 54,675 probe-sets, 27,535, 26,800 and 26,929 were scored as “present call” (neither marginal nor absent) in both PT and SAGM-grown cells of PT0808, PT0811 and PT0812, respectively. The tree view of hierarchical clustering using probes with present call indicated distinct patterns in gene expressions between PT and SAGM-grown cells (Figure 4A).

We next checked the fold-change of interested genes (Table 3). Probes with absent call in either PT or SAGM sample are also included because it is still possible to estimate the significant change even though its fold-change is not reliable. Stem cell marker *ABCG2*, *Oct-4* and *CD133* were not expressed in the SAGM-grown cells. Thyroid-specific genes such as *TG*, *TSH-R*, *PAX8*, *TTF1* and *TPO* seemed to be highly suppressed, which was validated by qRT-PCR (Figure 4B). Among other tissue stem cell markers, *CD106*, *CD105* and *CD90*, which are marker for mesenchymal and/or hematopoietic stem cell, were up-regulated. *E-cadherin*, whose loss is correlated with induction of EMT, was highly down-regulated.

**Table 3:** Fold-change of interested genes (SAGM cells/PT).

		PT0808			PT0811			PT0812		
Gene	Probe	PT-flag	SAGM-flag	fold	PT-flag	SAGM-flag	fold	PT-flag	SAGM-flag	fold
ABCG2	209735_at	A	A		A	A		A	A	
Oct4	208286_x_at	A	A		A	A		A	A	
CD133	204304_s_at	P	A	0.00	P	A	0.03	P	A	0.04
TG	203673_at	P	A	0.05	P	A	0.00	P	A	0.19
TSHR	210055_at	P	A	0.04	P	A	0.16	P	A	0.04
	215442_s_at	P	P	0.06	P	A	0.25	P	P	0.20
	215443_at	P	P	0.06	P	A	0.02	P	A	0.05
	237349_at	A	A		A	A		A	A	
PAX8	121_at	P	P	0.06	P	P	0.05	P	P	0.11
	207921_x_at	P	A	0.19	P	A	0.09	P	P	0.24
	207923_x_at	P	A	0.06	P	A	0.01	P	A	0.14
	207924_x_at	P	A	0.14	P	A	0.01	P	A	0.10
	209552_at	P	P	0.04	P	A	0.01	P	P	0.03
	213917_at	P	A	0.13	P	A	0.02	P	A	0.03
	214528_s_at	P	A	0.02	P	A	0.02	P	A	0.07
	221990_at	P	A	0.12	P	A	0.00	P	A	0.06
TPO	210342_s_at	P	A	0.34	P	A	0.03	P	A	0.62
TTF1	210673_x_at	P	A	0.01	P	A	0.02	P	A	0.44
	211024_s_at	P	P	0.04	P	P	0.04	P	P	0.12
CD106	203868_s_at	A	P	4.41	P	A	1.85	A	P	6.61
CD105	201808_s_at	A	A		A	A		A	A	
	201809_s_at	A	P	6.67	A	P	5.31	P	P	2.57
	228586_at	A	A		A	A		A	A	
CD90	208850_s_at	P	P	13.59	A	P	859.78	A	P	76.16
	208851_s_at	P	P	9.70	P	P	51.57	M	P	47.39
	213869_x_at	P	P	10.58	A	P	39.91	A	P	23.06
CD73	203939_at	P	P	0.83	P	P	1.27	P	P	1.50
	227486_at	P	P	1.22	P	P	1.46	P	P	1.18
	1553994_at	P	P	0.74	P	P	1.19	P	P	1.26
	1553995_a_at	P	P	0.70	P	P	1.16	P	P	1.24
KRT18	201596_x_at	P	P	0.02	P	P	0.02	P	P	0.31
Vim	201426_s_at	P	P	1.37	P	P	1.21	P	P	1.31
	1555938_x_at	P	P	0.75	P	P	1.00	P	P	0.46
E-CAD	201130_s_at	P	A	0.19	P	A	0.12	P	A	0.25
	201131_s_at	P	P	0.03	P	P	0.02	P	P	0.25

Flag score; P: present, M: marginal, A: absent.

Next, we selected 663 genes that were significantly (t-test, p<0.05) and >2.0-fold increased in all the SAGM-grown cells compared to corresponding PT. Likewise, 649 genes were selected as down-regulated genes (<0.5-fold). Probes with absent call in either PT or SAGM sample are excluded. To get insight into the biological significance, Gene Ontology (GO) enrichment analysis was performed to identify specifically regulated biological processes using the above selected genes. Sixteen and four GO processes were significantly impacted (p<0.1) in the up- and down-regulated gene sets, respectively (Table 4). Interestingly, some particular processes were impacted. Processes related to embryo implantation, endoplasmic reticulum, sterol/steroid, cell cycle and oxidoreductase were up-regulated, and those related to protein binding and cell adhesion were down-regulated (Table 4).

**Table 4:** Up- or down-regulated Gene Ontology (GO) cellular processes.

(>2.0-fold)		
GO Accession	GO Term	corrected p-value
GO:0007566	embryo implantation	1.08E-05
GO:0005788|GO:0016022	endoplasmic reticulum lumen	1.33E-05
GO:0006694	steroid biosynthetic process	4.30E-04
GO:0044432	endoplasmic reticulum part	4.30E-04
GO:0005783	endoplasmic reticulum	4.30E-04
GO:0016126	sterol biosynthetic process	8.81E-04
GO:0008202	steroid metabolic process	0.004734457
GO:0008610	lipid biosynthetic process	0.008595788
GO:0016125	sterol metabolic process	0.011360187
GO:0015630	microtubule cytoskeleton	0.028312529
GO:0044444	cytoplasmic part	0.036239438
GO:0000278	mitotic cell cycle	0.039986596
GO:0016614	oxidoreductase activity, acting on CH-OH group of donors	0.053449787
GO:0016616	oxidoreductase activity, acting on the CH-OH group of donors, NAD or NADP as acceptor	0.06185003
GO:0007049	cell cycle	0.063007735
GO:0005793	ER-Golgi intermediate compartment	0.073000684

(<0.5-fold)		
GO Accession	GO Term	corrected p-value
GO:0008092	cytoskeletal protein binding	2.59E-04
GO:0007156	homophilic cell adhesion	2.59E-04
GO:0005515|GO:0045308	protein binding	0.001197793
GO:0016337	cell-cell adhesion	0.004711885

## Discussion

In the present study, we have developed a novel system to trigger dedifferentiation of normal human thyroid follicular cells. We used a commercially available medium which allowed universal and reproducible procedure. We investigated the origin of the cells by analyzing expressions of STRO-1 and two intermediate filaments, cytokeratin-18 and vimentin. In our system, there were only two types of attached cells in terms of expression pattern of the above-mentioned markers after initial plating: cytokeratin-18/vimentin double-positive cells and STRO-1/vimentin double-positive cells. Thyroid follicular cells coexpress cytokeratin-18 and vimentin, which is consistent with earlier studies [18], [19], [20]. Cytokeratin-18/vimentin-positive cells may also contain a small number of endothelial cells since several papers demonstrated expression of cytokeratin in microvascular endothelial cells in different tissues [21], [22], [23], [24], [25]. STRO-1/vimentin-positive cells are perhaps composed of premature mesenchymal cells. The SAGM-grown cells were propagated from thyroid follicular cells (or at least thyroid-committed cells) because: (1) the SAGM-grown cells were negative for STRO-1; (2) the cells lost cytokeratin-18 expression during expansion; (3) STRO-1-positive cells did not proliferate; (4) *TG* mRNA expression was also decreased during proliferation but still detectable after 3–5 weeks; (5) the SAGM-grown cells were propagated from sorted TPO^hi^ cells.

Although both cytokeratin-18 and vimentin expressions were observed even in undifferentiated thyroid cancer cell lines, the cytokeratin-18 expression was lost in the SAGM-grown cells. All of thyroid-specific gene expressions were not observed in the cells. Moreover, the SAGM-grown cells displayed high plasticity: multilineage differentiation potential into thyrocytes, neuronal cells and adipocytes. These results suggest that we have successfully dedifferentiated/converted thyroid follicular cells into multilineage progenitor cells. However, re-differentiation effects (expression level of thyroid-specific genes) were modest compared to PT. We are currently seeking the better method enabling more efficient differentiation. In addition, these cells do not have unlimited proliferative capacity: the cells will stop growing until 3–4 months, due to cellular senescence.

Conversion of differentiated cells into multipotent progenitor or different lineage has been reported by several groups. Adult hepatocytes were converted into insulin-producing cells by transgenes, *Pdx-1* and *Ngn-3* [26]; retinal pigment epithelium into retinal neurons by *Sox-2* [27]; and interfollicular epidermal basal keratinocytes into the cells capable of differentiating into neuronal cells by *Oct-4* [28]. The aforementioned studies used exogenous trangenes, some of which are key transcription factors for generating iPS cells. To our knowledge, there is only one report describing conversion from differentiated cells into multilineage progenitor cells without any gene delivery. Adult intestinal epithelial cells were dedifferentiated into nestin-positive cells that have multilineage differentiation capacity into neuronal, pancreatic and hepatic lineages [29]. The authors cultured intestinal epithelial cells on mouse embryonic fibroblasts (feeder layer) in medium supplemented with leukemia inhibitory factor, EGF and bFGF. For generating iPS cells, two key transcription factors Klf-4 and Sox-2 could be replaced by chemical compounds [30], [31]. SAGM contains EGF, insulin, other growth factors and chemicals. These findings suggest that a particular combination of growth factors/chemicals probably changes transcriptional profiling, leading to dedifferentiation of thyrocytes.

Microarray analysis revealed that the expression of *ABCG2*, *Oct-4* and *CD133* which have been used to identify stem cells in a number of tissues, were not up-regulated in the SAGM-grown cells, suggesting that the cells were a bit far from genuine pluripotent stem cells. Rather, they seem to be more committed stem/progenitor cells. In fact, mesenchymal stem cell markers *CD106*, *CD105* and *CD90* but not *CD73* were highly up-regulated. Taken together with down-regulation of *E-cadherin*, these results suggest that the SAGM-grown cells acquired properties prior to cells committed to thyroid epithelial lineage.

GO enrichment analysis was performed and revealed differentially regulated cellular processes. Embryo implantation had the lowest p value among the up-regulated GO processes. Other up-regulated processes were endoplasmic reticulum, sterol/steroid/lipid biosynthesis/metabolic processes, cell cycle and oxidoreductase activity. These processes are generally related to protein/membrane synthesis presumably linked to cell proliferation. Regarding down-regulated GO processes, protein binding and cell adhesion processes were impacted. The down-regulation of cytoskeletal protein binding process might reflect to the loss of cytokeratin-18 expression. The reduction of genes of cell adhesion process might be a consequence of the loss of epithelial property and/or polarity. It should be noted that we selected only probes scored as “present call” in all samples, which allows relatively accurate comparison of expression levels between samples. However, this means that genes with very low expression in either PT or the SAGM-grown cells were probably excluded even though their difference in expression levels was far greater.

In summary, we have developed a novel system to propagate multilineage progenitor cells from adult normal human thyroid tissues. This seems to be achieved by dedifferentiation of thyroid follicular cells without any gene delivery. Since integration of transgene(s) may cause unpredictable problem, our system has an advantage in terms of safety. The presently described culture system may be useful for regenerative medicine, but the primary importance will be as a tool to elucidate the progression of thyroid disease. Moreover, this phenomenon could be induced *in vivo* because it can be achieved without introducing foreign genes. However, as we have not confirmed full functional differentiation of the cells, further study is necessary for regenerative application.

## Supporting Information

Figure S1 Asymmetric division of the SAGM-grown cells. A, Scheme of cell divisions. Representative data obtained by time-lapse imaging of cell cycle are shown. ACD: asymmetric cell division, SCD: symmetric cell division. 6.1% of the cells showed asymmetric division after first division. Total 198 cells were analyzed. B, Representative images after asymmetric division.
(TIF)

Table S1 Time-course expression of lineage-specific markers.
(PDF)

Table S2 Colony formation in SAGM after FACS.
(PDF)
