# Supplementary figures and images for: Dedifferentiation of Human Primary Thyrocytes into Multilineage Progenitor Cells without Gene Introduction
Source: PLoS One. 2011 Apr 27;6(4):e19354. doi: 10.1371/journal.pone.0019354 (PMC3083435; doi:10.1371/journal.pone.0019354)

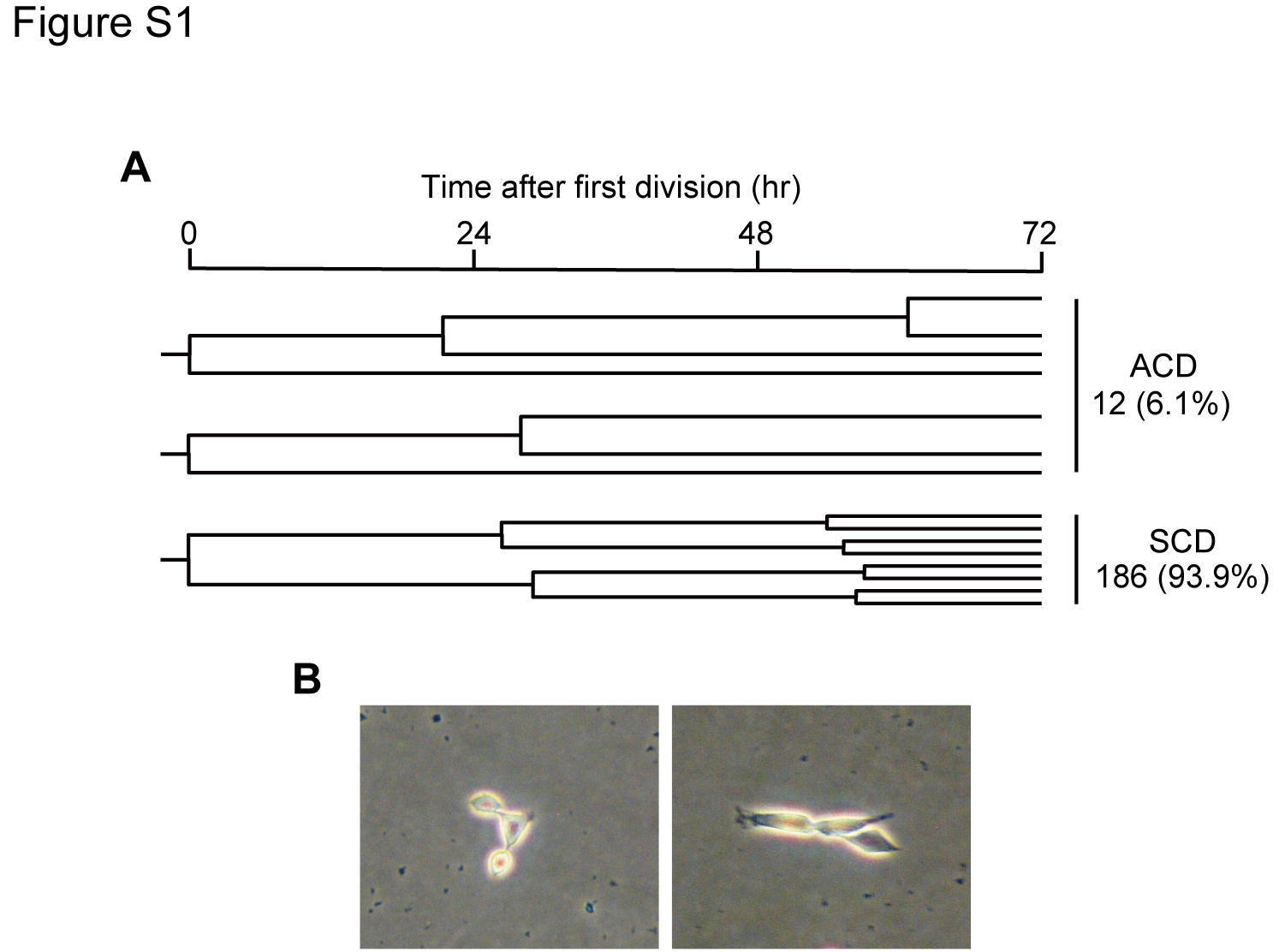

Supplement: Figure S1 — Asymmetric division of the SAGM-grown cells. A, Scheme of cell divisions. Representative data obtained by time-lapse imaging of cell cycle are shown. ACD: asymmetric cell division, SCD: symmetric cell division. 6.1% of the cells showed asymmetric division after first division. Total 198 cells were analyzed. B, Representative images after asymmetric division. (TIF) [file pone.0019354.s001.tif]
