# Supplementary material for: Dedifferentiation of Human Primary Thyrocytes into Multilineage Progenitor Cells without Gene Introduction
Source: PLoS One. 2011 Apr 27;6(4):e19354. doi: 10.1371/journal.pone.0019354 (PMC3083435; doi:10.1371/journal.pone.0019354)
Supplement: Table S1 — Time-course expression of lineage-specific markers. (PDF) [file pone.0019354.s002.pdf]

Table S1. Time-course expression of lineage-specific markers.

PT-0811	Total count	Cyt^{+}	Cyt^{+}/TG^{+}	$Vim^{+}/STRO\text{-}1^{+}$
Day 1	2790	2763 (99.0)	2763 (99.0)	25 (0.9)
Day 5	2661	2661 (93.2)	982 (36.9)	21 (0.8)
Day 8	2679	873 (32.6)	239 (8.9)	11 (0.4)
Day 10	3032	726 (23.9)	27 (0.9)	7 (0.2)

No. of cells (%)

Determined by immunofluorescence.
