# Supplementary material for: Dedifferentiation of Human Primary Thyrocytes into Multilineage Progenitor Cells without Gene Introduction
Source: PLoS One. 2011 Apr 27;6(4):e19354. doi: 10.1371/journal.pone.0019354 (PMC3083435; doi:10.1371/journal.pone.0019354)
Supplement: Table S2 — Colony formation in SAGM after FACS. (PDF) [file pone.0019354.s003.pdf]

Table S2. Colony formation in SAGM after FACS.

Cells	Sorted STRO-1^{+}	Sorted TPO^{hi}	colony
PT-0905	50		-
		2,000	+
PT-1001	100		-
		5,000	+
PT-1002	500		-
		100,000	+++
PT-1003	100		-
		10,000	++
